# Serum levels of mitochondrial inhibitory factor 1 are independently associated with long-term prognosis in coronary artery disease: the GENES Study

**DOI:** 10.1186/s12916-016-0672-9

**Published:** 2016-08-23

**Authors:** Annelise Genoux, Laeticia Lichtenstein, Jean Ferrières, Thibaut Duparc, Vanina Bongard, Paul-Louis Vervueren, Guillaume Combes, Dorota Taraszkiewicz, Meyer Elbaz, Michel Galinier, Bertrand Nassar, Jean-Bernard Ruidavets, Bertrand Perret, Laurent O. Martinez

**Affiliations:** 1 Institute of Metabolic and Cardiovascular Diseases, I2MC, Inserm, UMR 1048, Toulouse, France; 2 Université de Toulouse, UMR1048, Toulouse, France; 3 CHU Toulouse, Toulouse University Hospital, Service de Biochimie, Pôle biologie, Hôpital de Purpan, Toulouse, France; 4 Department of Epidemiology, Health Economics and Public Health, Inserm, Université de Toulouse, CHU Toulouse, UMR1027, Toulouse, France; 5 CHU Toulouse, Toulouse University Hospital, Fédération de Cardiologie, Toulouse, France

**Keywords:** High density lipoprotein, HDL, Cholesterol, ATP synthase, F_1_-ATPase, Inhibitory Factor 1, IF1, Biomarker

## Abstract

**Background:**

Epidemiological and observational studies have established that high-density lipoprotein cholesterol (HDL-C) is an independent negative cardiovascular risk factor. However, simple measurement of HDL-C levels is no longer sufficient for cardiovascular risk assessment. Therefore, there is a critical need for novel non-invasive biomarkers that would display prognostic superiority over HDL-C. Cell surface ecto-F_1_-ATPase contributes to several athero-protective properties of HDL, including reverse cholesterol transport and vascular endothelial protection. Serum inhibitory factor 1 (IF1), an endogenous inhibitor of ecto-F_1_-ATPase, is an independent determinant of HDL-C associated with low risk of coronary artery disease (CAD). This work aimed to examine the predictive value of serum IF1 for long-term mortality in CAD patients. Its informative value was compared to that of HDL-C.

**Method:**

Serum IF1 levels were measured in 577 male participants with stable CAD (age 45–74 years) from the GENES (Genetique et ENvironnement en Europe du Sud) study. Vital status was yearly assessed, with a median follow-up of 11 years and a 29.5 % mortality rate. Cardiovascular mortality accounted for the majority (62.4 %) of deaths.

**Results:**

IF1 levels were positively correlated with HDL-C (*r*_s_ = 0.40; *P* < 0.001) and negatively with triglycerides (*r*_s_ = −0.21, *P* < 0.001) and CAD severity documented by the Gensini score (*r*_s_ = −0.13; *P* < 0.01). Total and cardiovascular mortality were lower at the highest quartiles of IF1 (HR = 0.55; 95 % CI, 0.38–0.89 and 0.50 (0.28–0.89), respectively) but not according to HDL-C. Inverse associations of IF1 with mortality remained significant, after multivariate adjustments for classical cardiovascular risk factors (age, smoking, physical activity, waist circumference, HDL-C, dyslipidemia, hypertension, and diabetes) and for powerful biological and clinical variables of prognosis, including heart rate, ankle-brachial index and biomarkers of cardiac diseases. The 10-year mortality was 28.5 % in patients with low IF1 (<0.42 mg/L) and 21.4 % in those with high IF1 (≥0.42 mg/L, *P* < 0.02).

**Conclusions:**

We investigated for the first time the relation between IF1 levels and long-term prognosis in CAD patients, and found an independent negative association. IF1 measurement might be used as a novel HDL-related biomarker to better stratify risk in populations at high risk or in the setting of pharmacotherapy.

**Electronic supplementary material:**

The online version of this article (doi:10.1186/s12916-016-0672-9) contains supplementary material, which is available to authorized users.

## Background

A low level of high density lipoprotein-cholesterol (HDL-C) is a robust and independent risk factor of coronary artery diseases (CAD) as it represents the cholesterol content of HDL particles [1–4]. To date, however, it has proved difficult to successfully reduce cardiovascular risk with HDL-C raising drugs such as niacin or inhibitors of cholesteryl ester transfer protein [5–7]. Furthermore, Mendelian randomization studies of common genetic variants for HDL-C are inconsistent in their support of a causal relationship between HDL-C level and CAD [8]. These observations underline the potential limitation of using HDL-C levels (a static mass based measurement) for assessing CAD risk or for evaluating the therapeutic efficiency of treatments aimed at HDL. In fact, only 5 % of the total HDL-C is derived from actual macrophage cholesterol efflux [9], indicating that HDL-C is a poor surrogate of reverse cholesterol transport (RCT), the postulated main anti-atherogenic effect of HDL. Further, HDL cholesterol content does not represent other important anti-atherogenic HDL properties, such as anti-inflammatory, anti-thrombotic, anti-oxidant, vasorelaxant, and cytoprotective effects [10, 11]. Thus, a great clinical need exists for HDL-related biomarkers that, more directly than HDL-C (HDL-C “quantity”), reflect the functionality of HDL (HDL “quality”) [11].

A few years ago, while studying the last steps of RCT, we identified plasma membrane ecto-F_1_-ATPase as a high affinity receptor for HDL apolipoprotein A-I (apoA-I), triggering HDL uptake by the liver [12]. More recently still, ecto-F_1_-ATPase was identified to also be an apoA-I receptor involved in HDL-mediated endothelial cell survival and HDL transcytosis through aortic endothelial cells [13, 14]. Ecto-F_1_-ATPase is identical to the catalytic F_1_ domain of the mitochondrial F_1_F_O_-ATP-synthase, which synthesizes ATP in response to proton flux from the inter-membrane space to the mitochondrion matrix [15]. In acidosis conditions, when the proton flux is impaired, the ATP-synthase complex turns into an ATP-hydrolytic enzyme. A mitochondrial peptide, inhibitory factor 1 (IF1), then binds to the complex, inhibiting its hydrolytic activity and preventing the breakdown of the ATP in cellular pools [15]. Experimentally, exogenous IF1 can also inhibit the ATPase activity of plasma membrane ecto-F_1_-ATPase and block HDL endocytosis in hepatocytes and endothelial cells [12, 14].

Unexpectedly, we have recently found IF1 to be present in human serum, its concentration following a normal distribution in the general population [16]. Circulating IF1 was found to be associated with HDL-C, suggesting it could be a biomarker indicative of F_1_-ATPase-mediated HDL functions [16, 17]. In the context of a large case-control study on CAD, the *GENES* study, IF1 was found to be negatively associated with CAD [17]. This association was independent of HDL-C and of other classical cardiovascular risk factors [17]. These conclusions were drawn from association studies in a cross-sectional design. However, the vital status of all stable CAD patients from this cohort was available with a median follow-up of 11 years. Therefore, the aim of the present study was to investigate the association of IF1 serum level with mortality in CAD patients from the *GENES* study. This association was compared to that of HDL-C.

## Methods

### Study participants

The Genetique et ENvironnement en Europe du Sud (GENES) study is a case-control study designed to investigate the role of gene–environment interactions in the occurrence of CAD [18]. From 2002 to 2004, we have prospectively recruited 834 male patients aged between 45 and 74 years, living in the Toulouse area (south-western France), admitted in the department of Cardiology of the Toulouse University Hospital, and referred for evaluation and management of stable CAD. Stable CAD was defined by a previous history of acute coronary syndrome, a previous history of coronary artery revascularization, a documented myocardial ischemia, a stable angina, or the presence at the coronary angiography of a coronary stenosis of ≥ 50 % of luminal narrowing. Patients who had an acute coronary episode during the past 7 days were not included in the study because they were not considered as stable. In the present analysis, we only took into account the first 577 patients in whom total serum IF1 was measured and complete data were available.

### Assessment of the vital status

Vital status on December 31, 2013, was obtained for each participant through the national database (“RNIPP”), which records annual all deaths occurring in the French population (http://cesp.vjf.inserm.fr/svcd). Vital status was assessed yearly, with a median follow-up of 11 years. All dates and causes of death were obtained for participants who died during the follow-up period. Main and associated causes of death were provided by the French National Institute of Health Research (CépiDc-INSERM), which systematically collects and codes (using the International Classification of Diseases coding system) data recorded on death certificates. Death from a cardiovascular cause during follow-up was assessed by a committee of four medical doctors, every time cardiovascular disease was reported as the main cause of death, or when it was mentioned as an associated cause, if the main cause was a plausible complication of cardiovascular disease. Authorizations to use these data were obtained in accordance with French law (Commission nationale de l'informatique et des libertés (CNIL): authorization 355152v1, September 3, 2008).

### Measured parameters

Age, environmental characteristics, and information on cardiovascular risk factors were collected through standardized face-to-face interviews, performed by a single physician. Smoking status was classified as current smokers, smokers having quit for more than 3 years, and non-smokers. Among current smokers, cigarette consumption was estimated with the pack-year quantification and recorded as the average number of cigarettes per day. Alcohol consumption was assessed using a typical week pattern. The total amount of pure alcohol consumption was calculated as the sum of different types of drinks and was expressed as grams per day. Physical activity was investigated through a standardized questionnaire [19] and categorized into three levels as no physical activity, moderate physical activity during 20 minutes no more than once a week, and high physical activity during 20 minutes, at least twice a week. Presence of dyslipidemia, diabetes mellitus or hypertension was assessed from the subjects’ current treatments. Past medical history was collected and, for cases, was also checked in the patients’ medical files. Medications at discharge were also considered in patients.

A trained physician performed physical examinations, using standard measurement methods. Anthropometrical measurements including waist circumference, height, and body weight were performed and body mass index (BMI, kg/m^2^) was calculated. Blood pressure and resting heart rate were measured with an automatic sphygmomanometer (OMRON 705 CP). Measurements were performed after a minimum of 5 minutes rest; average values from two different measurements were recorded for further analysis. Ankle-brachial index was determined as previously described, and a value of ≤ 0.9 was considered abnormal [20].

### Assessment of CAD severity and extension and estimation of cardiac function

Coronary artery stenoses of ≥ 50 % luminal narrowing were considered significant. Diffusion of coronary artery disease lesions were assessed by calculating the Gensini Score, based on data from coronary angiography [21–23]. Left ventricular ejection fraction (LVEF) was assessed by contrast ventriculography using an isotopic method, and/or by echocardiography.

### Laboratory assays

Blood was collected after an overnight fast. Serum sample aliquots were subsequently stored at −80 °C until biological analyses. The following biomarkers were assayed with enzymatic reagents on automated analyzers (Hitachi 912 and Cobas 8000, Roche Diagnostics, Meylan, France): serum total cholesterol, HDL-C, triglycerides, fasting glucose, high-sensitivity C reactive protein (hs-CRP), N-terminal pro-brain natriuretic peptide (NT-proBNP), and high-sensitivity cardiac troponin T (hs-TnT). Lipoprotein(a) was assayed with an immunoturbidimetric method on an automated analyzer (Roche Diagnostics, Meylan, France) and estimated glomerular filtration rate (eGFR) was calculated using the abbreviated Modification of Diet in Renal Disease Study equation [18]. Serum IF1 concentration was measured using a competitive ELISA immuno-assay previously developed in our laboratory [16] and for which intra-assay and inter-assay variability ranged from 6 % to 7 % and several other tests were performed including absence of interference with apolipoproteins A-I and A-II or resistance to freezing and thawing [17].

### HDL measurement by nuclear magnetic resonance (NMR) spectroscopy

HDL particle concentration and size were measured by NMR spectroscopy using the AXINON® lipoFIT®-S100 test system (Numares AG, Regensburg, Germany) in 212 randomly selected CAD patients from the initial sample. Briefly, ^1^H NMR spectra were recorded at a temperature of 310 K on a shielded 600 MHz Avance III HD NMR spectrometer (Bruker Biospin) with a 5 mm triple resonance TXI probe head including a deuterium lock channel, atm unit, and a z-gradient coil. Lipoprotein analysis was conducted via deconvolution of the broad methyl group signal at approximately 0.9–0.8 ppm, delivering the concentrations of lipoprotein particles as well as the average particle size. In addition, a custom algorithm was used to mathematically remove a signal derived from gel separator blood collection tubes interfering with the calculation. This algorithm is not part of the CE-marked lipoFIT®-S100 software. In this study, the concentrations of total HDL particles (reported in μmol/L) as well as the average HDL particle size (reported in nm) are used.

### Statistical analyses

Continuous variables are displayed as means and standard deviations. Categorical variables are presented as proportions. We first described and compared the characteristics of participants according to vital status. Categorical variables were compared between groups using the χ^2^ test (or Fisher’s exact test when necessary). The Student’s *t* test was used to compare the distribution of continuous data. A Mann–Whitney’s test (or logarithmic transformation of the variable when necessary) was performed when distribution departed from normality, or when homoscedasticity was rejected. Spearman rank correlations were used to test the associations of IF1 and HDL-C with cardiovascular risk factors, severity, extension, and estimation of cardiac function of the disease.

Cumulative survival of patients were determined by the Kaplan–Meier method and compared using the Log-rank test for the individual endpoints of all-cause mortality. The relation between baseline variables and mortality was assessed using Cox proportional hazards regression analysis. We tested the proportionality assumption using cumulative sums of martingale-based residuals. We performed regression analyses with polynomial models (quadratic and cubic) to examine for possible non-linear relations between continuous variables and mortality. Cox regression analyses were performed first without any adjustment for co-variables and, second, with adjustment on classical cardiovascular risk factors (age, treatments for dyslipidemia, hypertension and diabetes, smoking and HDL-C) and extended cardiovascular risk factors (classical risk factors plus waist circumference, physical activity, CRP, eGFR, and duration of CAD). Further adjustments were successively performed on other biomarkers of cardiac diseases (NT-proBNP and hs-TnT) as well as non-invasive and invasive clinical parameters related to the severity and extension of the disease and cardiac function (heart rate, ABI, LVEF and Gensini score). Finally, the association of mortality with IF1 according to median cutoff of serum concentration was analyzed. All statistical analyses were carried out using the SAS statistical software package 9.4 (SAS Institute, Cary, NC). All tests were considered significant at a *P* value < 0.05.

## Results

### Bio-clinical parameters according to vital status

In the *GENES* study, a cohort of 832 CAD patients was constituted between 2002 and 2004. IF1 measurements were available for a subsample of the first 651 patients from this initial cohort. During follow-up, the vital status of patients was assessed yearly. The median follow-up period was 11 years (mean: 9.9 years). Complete clinical and biological data, including IF1 measurements, were available for 577 patients (70 % of the whole cohort). Among them, by December 2013, 170 deaths had been recorded during follow-up, giving a mortality rate of 29.5 %, similar to that observed in the whole cohort (29.6 %), and a mean annual death rate of 2.9 % (95 % CI, 1.6–4.4; *P* < 0.01). Cardiovascular mortality accounted for the majority (62.4 %) of deaths recorded, whereas cancers accounted for 17.1 %. Other causes amounted to 20.5 %.

There were noticeable differences between cohort patients who died and those who survived during follow-up in the values for data determined at the start of the study (Table 1). Patients who died were older and had suffered with CAD for longer. They tended to be of a lower educational level and to have been less physically active. Among classical risk factors, tobacco consumption and treatment for diabetes were more frequent in patients who subsequently died than in survivors and average fasting glycaemia tended to be higher. Conversely, lipid-lowering therapy was more frequent in patients that survived (Table 1). Statins had been administered to 62.2 % of surviving patients versus 51.2 % of those who had died (Additional file 1: Table S1). Systolic blood pressure was not different between the two groups (Table 1). Beta-blockers and anti-platelet agents (acetylsalicylic acid or clopidogrel) were more frequently administered among surviving patients than in deceased ones (Additional file 1: Table S1), probably reflecting treatment efficacy. The reverse situation was observed for angiotensin converting enzyme (ACE) inhibitors, more frequently found in deceased patients. ACE inhibitors are particularly administered to patients presenting signs of congestive heart failure; our data may indicate a higher prevalence of this condition among patients having deceased.

**Table 1:** Clinical and biological characteristics in coronary artery disease patients when they were first included in the GENES study

	Alive (*n* = 407)	Dead (*n* = 170)	*P*
Age, years	59.5 (7.8)	62.9 (8.0)	0.03
School, years of education	9.7 (3.0)	9.2 (2.6)	0.03^b^
Smoking, pack year	31.3 (31.1)	49.3 (44.1)	0.001^b^
Alcohol, g/day	28.4 (31.6)	28.7 (29.6)	0.93
Physical activity (high level), %	14.3	5.3	0.003
Treatment for diabetes, %	19.4	36.5	0.001
Treatment for dyslipidemia, %	67.6	56.5	0.02
Treatment for hypertension, %	42.7	50.0	0.12
BMI, kg/m^2^	27.3 (3.8)	27.7 (4.4)	0.27
Waist circumference, cm	97.9 (10.0)	101.5 (12.7)	0.001
Systolic blood pressure, mm Hg	139.2 (20.1)	139.8 (22.0)	0.77
Heart rate, beats/min	62.2 (10.6)	67.1 (13.3)	0.001^a^
Fasting glucose, mmoL/L	5.69 (1.58)	6.41 (2.72)	0.06^b^
Triglycerides, g/L	1.66 (0.88)	1.57 (0.80)	0.17^a^
Total cholesterol, g/L	2.01 (0.41)	1.94 (0.43)	0.06
LDL-C, g/L	1.25 (0.35)	1.20 (0.39)	0.13
HDL-C, g/L	0.43 (0.12)	0.43 (0.11)	0.42
Lipoprotein(a) ≥ 0.30 vs. < 0.30 g/L, %	55.8	47.7	0.08
eGFR < 30 mL/min, %	0.5	4.7	0.002^c^
hs-CRP ≥ 5 mg/L vs. < 5 mg/L, %	51.6	64.1	0.006
hs-TnT, pg/mL	145.9 (386.2)	198.1 (469.1)	0.002^b^
NT-proBNP, pg/mL	383.3 (683.1)	1253.9 (2347.5)	0.001^b^
ABI ≤ 0.9, %	29.7	47.1	0.001
LVEF ≥ 50 %, %	77.2	49.4	0.001
Gensini score	43.8 (37.3)	57.3 (46.3)	0.003^a^
Duration of coronary artery disease, months	33.4 (57.2)	57.6 (73.2)	0.001^b^
IF1, mg/L	0.44 (0.12)	0.41 (0.13)	0.03

Data are expressed in mean (SD) or %

*BMI* body mass index, *eGFR* estimated Glomerular Filtration Rate, *hs-CRP* high-sensitivity C-Reactive Protein, *hs-TnT* high-sensitivity cardiac troponin T, *NT-proBNP* N-terminal pro-brain natriuretic peptide, *ABI* Ankle-brachial index, *LVEF* Left Ventricular Ejection Fraction

^a^log transformed data, ^b^Wilcoxon–Mann–Whitney test, ^c^Fischer’s exact test

Among the clinical variables, mortality was associated with higher waist circumference, higher heart rate, and a higher proportion of a pejorative ankle-brachial index (ABI), but also with lower LVEF, since only 49 % of dead patients had LVEF ≥ 50 % versus 77 % in survivors. Lesion severity, as documented by the angiographic Gensini score, was more pronounced in deceased patients. Among metabolic parameters, LDL-C or HDL-C were not different between the two groups but patients who died had lower IF1 concentrations. IF1 concentrations were not significantly different between statin-treated or non-treated patient subgroups (interaction statin × IF1, *P* = 0.68), either deceased or still alive (not shown).

NT-proBNP and hs-TnT, two biomarkers associated with myocardial damages, were higher in patients who died. An elevated hs-CRP, documenting an inflammatory condition, was more frequent among deceased patients. Finally, severe renal failure, as documented by a glomerular filtration rate below 30 mL/min, was more frequent in deceased patients.

### Correlations between IF1, HDL-C, cardiovascular risk factors, and variables

As commonly reported, HDL-C was found to be negatively correlated with several different cardiovascular risk markers. In particular, there was a negative correlation with those markers associated with metabolic syndrome: triglycerides, systolic blood pressure, BMI, and waist circumference (Table 2). HDL-C was also inversely related to CAD severity, as documented by the Gensini score. HDL-C was positively associated with alcohol consumption and physical activity and negatively associated to cardiac biomarkers indicative of myocardial damages (NT-proBNP and hs-TnT) and to hs-CRP, reflecting an inflammatory condition.

**Table 2:** Correlation between IF1, HDL-C, and other cardiovascular risk factors in coronary artery disease patients

	IF1 (mg/L)	HDL-C (g/L)
Age, years	−0.10 (−0.18 to −0.02)^*^	0.10 (0.02 to 0.18)^*^
Smoking, cig/day	0.02 (−0.05 to 0.11)	−0.08 (−0.16 to 0.01)
Alcohol, g/day	0.03 (−0.05 to 0.11)	0.16 (0.08 to 0.23)^***^
Physical activity score	0.15 (0.07 to 0.23)^***^	0.13 (0.05 to 0.21)^**^
BMI, kg/m^2^	−0.05 (−0.13 to 0.04)	−0.17 (−0.25 to −0.09)^***^
Waist circumference, cm	−0.09 (−0.17 to −0.01)^*^	−0.16 (−0.24 to −0.08)^***^
SBP, mm Hg	0.01 (−0.09 to 0.08)	0.12 (0.04 to 0.20)^***^
Heart rate, beats/mn	−0.07 (−0.16 to −0.01)	0.03 (−0.05 to 0.11)
Glucose, mmoL/L	−0.01 (−0.9 to 0.07)	−0.11 (−0.19 to −0.02)^*^
Triglycerides, g/L	−0.21 (−0.28 to −0.13)^***^	−0.41 (−0.48 to −0.34)^***^
Total cholesterol, g/L	0.08 (0.01 to 0.16)^*^	0.22 (0.14 to 0.30)^***^
HDL-C, g/L	0.40 (0.33 to 0.46)^***^	
eGFR, mL/min	0.09 (0.01 to 0.17)^*^	−0.01 (−0.08 to 0.08)
hs-CRP, mg/L	−0.14 (−0.22 to −0.06)^***^	−0.19 (−0.27 to −0.11)^***^
hs-TnT, pg/ml	−0.07 (−0.15 to 0.01)	−0.15 (−0.23 to −0.07)^***^
NT-proBNP, pg/mL	−0.18 (−0.26 to −0.10)^***^	−0.11 (−0.18 to −0.02)^*^
LVEF, %	0.19 (0.11 to 0.27)^***^	0.05 (−0.03 to 0.14)
Gensini score	−0.13 (−0.20 to −0.04)^**^	−0.15 (−0.23 to −0.07)^***^

Spearman rank correlation (95 % confidence interval).^*^ *P* < 0.05, ^**^ *P* < 0.01, ^***^ *P* < 0.001

*BMI* body mass index, *SBP* systolic blood pressure, *hs-CRP* high-sensitivity C-Reactive Protein, *hs-TnT* high-sensitivity cardiac troponin T, *NT-proBNP* N-terminal pro-brain natriuretic peptide, *LVEF* left ventricular ejection fraction

Serum IF1 was positively correlated with HDL-C and negatively with triglycerides (Table 2), as we have previously reported in control subjects taken from the general population [17]. However, unlike HDL-C, IF1 was not correlated with blood pressure and alcohol consumption (Table 2). IF1 was negatively associated with hs-CRP and NT-proBNP, but not with hs-TnT. A positive association with LVEF and a negative one with the severity of CAD (Gensini score) were also recorded. A negative association with heart rate was observed close to significance (Table 2). Furthermore, IF1 was also related to eGFR. Thus, although correlated to HDL-C, IF1 displays specific associations with clinical variables.

The concentrations of total HDL particles (HDL-P, reported in μmol/L) as well as the average HDL particle size (reported in nm) were measured in 212 randomly selected CAD patients from the initial sample (mean, 26.43 μmol/L (SD 5.5) and 8.87 nm (SD 0.34), respectively). As reported in Additional file 1: Table S2, IF1 displays a positive association with HDL-C (*r*_s_ = 0.35, *P* < 0.001), similar to that observed in the first 577 CAD patients (*r*_s_ = 0.40, *P* < 0.001, Table 2). Interestingly, IF1 was moderately positively correlated with HDL-P and HDL particle size (Additional file 1: Table S2, *r*_s_ = 0.30, *P* < 0.001 and *r*_s_ = 0.21, *P* < 0.01, respectively). HDL-C was even more strongly positively associated with those NMR HDL measurements (*r*_s_ = 0.72, *P* < 0.001 and *r*_s_ = 0.48, *P* < 0.001, respectively). Finally, HDL-P was not correlated with HDL particle size (*r*_s_ = −0.04, *P* = 0.53), as previously reported in other studies [24, 25].

### Association of IF1 with mortality in CAD patients

The values of HDL-C and IF1 were divided into quartiles in order to test their impact on mortality that had occurred from any cause at any time during follow-up. No difference in death rates was seen across the quartiles of HDL-C distribution (Table 3). Conversely, death rates declined from 36.7 % to 22.9 % between quartiles 1 and 4 of IF1 distribution (*P* = 0.007). In the IF1 upper quartile, the hazard ratio (HR) was 0.55 (95 % CI, 0.38–0.89) for total mortality and 0.50 (95 % CI, 0.28–0.89) for cardiovascular mortality (Table 3). IF1 level was not associated to cancer mortality (Table 3).

**Table 3:** Death rate according to HDL-C and IF1 and association with mortality

HDL-C					
	Q1	Q2	Q3	Q4	*P* for trend
	(n = 145)	(n = 151)	(n = 132)	(n = 143)	
Death rates, %	25.5	34.4	29.5	29.4	0.65
Hazard ratio for total mortality, 95 % CI	1	1.42 (0.93–2.17)	1.18 (0.75–1.84)	1.18 (0.76–1.83)	0.72
Hazard ratio for cardiovascular mortality, 95 % CI	1	1.45 (0.88–2.57)	1.12 (0.60–2.07)	0.95 (0.50–1.80)	0.67
IF1					
	Q1	Q2	Q3	Q4	*P* for trend
	(n = 147)	(n = 141)	(n = 145)	(n = 144)	
Death rates, %	36.7	31.2	26.9	22.9	0.007
Death rate per-person years, number of deaths per 1000 person-years	38.8	32.2	27.2	23.2	0.007
Hazard ratio for total mortality, 95 % CI	1	0.83 (0.56–1.23)	0.69 (0.45–1.04)	0.55 (0.38–0.89)	0.008
Hazard ratio for cardiovascular mortality, 95 % CI	1	0.51 (0.28–0.94)	0.65 (0.38–1.12)	0.50 (0.28–0.89)	0.03
Hazard ratio for cancer mortality, 95 % CI	1	1.24 (0.51–2.99)	0.53 (0.18–1.57)	0.53 (0.18–1.58)	0.12

Quartiles for HDL-C: 0.38, 0.42, 0.48 g/L

Quartiles for IF1: 0.35, 0.41, 0.49 mg/L

In multivariable analyses, adjustments were made for multiple clinical or biological variables associated with mortality. Table 4 illustrates the HR of IF1 quartiles regarding total mortality, using different models. Association of elevated IF1 with decreased mortality was significant after adjustments on classical cardiovascular risk factors, including age, smoking, and treatments for dyslipidemia, hypertension, and diabetes (model 1, HR 0.61 (95 % CI, 0.39–0.94) for upper quartile). This remained true even after introduction of obesity, physical activity, HDL-C, hs-CRP, glomerular filtration rate, and disease duration (model 2, HR 0.54 (95 % CI, 0.33–0.88) for upper quartile). Further adjustments on cardiac biomarkers (model 3, HR 0.57 (95 % CI, 0.35–0.92)) or on clinical markers associated with cardiovascular mortality (model 4, HR 0.61 (95 % CI, 0.37–0.99)) had little effects on the association between IF1 and mortality. Similar trends were observed with cardiovascular mortality (not shown). Although analyses are presented using quartile distribution of IF1, significant associations with mortality were also evident when considering IF1 as a continuous variable. Indeed, the unadjusted HR for total mortality per 1-SD increase in IF1 was 0.84 (95 % CI, 0.71–0.98). Similar values were measured after adjustments on classical risk factors (HR 0.83, 95 % CI, 0.69–0.99).

**Table 4:** Risk of mortality as a function of IF1 levels in coronary artery disease patients

	Q1	Q2	Q3	Q4
	(n = 147)	(n = 141)	(n = 145)	(n = 144)
		HR (95 % CI)	HR (95 % CI)	HR (95 % CI)
Model 1	1	0.88 (0.59–1.32)	0.77 (0.51–1.16)	0.61 (0.39–0.94)
Model 2	1	0.90 (0.59–1.36)	0.70 (0.45–1.08)	0.54 (0.33–0.88)
Model 3	1	0.81 (0.53–1.24)	0.73 (0.47–1.12)	0.57 (0.35–0.92)
Model 4	1	1.03 (0.67–1.56)	0.77 (0.50–1.20)	0.61 (0.37–0.99)

Quartiles for IF1: 0.35, 0.41, 0.49 mg/L

HR (95 % CI): Hazard Ratio (95 % confidence interval)

Model 1: adjusted for classical cardiovascular risk factors (age, smoking, treatments for dyslipidemia, hypertension and diabetes)

Model 2: adjusted for extended cardiovascular risk factors (Model 1 + physical activity, waist circumference, HDL-C, hs-CRP and eGFR and duration of CAD)

Model 3: model 2 plus NT-proBNP and hs-TnT

Model 4: model 2 plus heart rate and ankle-brachial index

### Survival of CAD patients as a function of IF1 concentrations

**Fig. 1:**
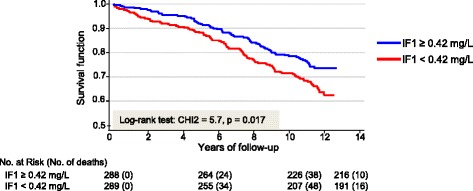
Kaplan–Meier survival curves. Survival curves for the follow-up period were established around the median value of serum IF1 concentration (0.42 mg/L)

Kaplan–Meier survival curves for the follow-up period were established around the median value of serum IF1 concentration (Fig. 1). Differences in mortality rates were evident depending on IF1 levels. After 10 years, mortality rate was 28.5 % in patients with low IF1 (<0.42 mg/L) and 21.4 % in those with high IF1 (*P* < 0.02).

## Discussion

In the present study, an average follow-up of 11 years of the vital status of CAD patients revealed that high circulating levels of IF1, but not HDL-C, are negatively associated with all-cause and cardiovascular disease mortality. For IF1, this remained true even after adjustments for multiple cardiovascular risk factors associated with mortality, including HDL-C and biomarkers of cardiac diseases such as NT-proBNP and Hs-TnT.

The fact that HDL-C was not associated with mortality is consistent with other studies showing that the association of HDL-C with cardiovascular mortality is weakened in patients with CAD [26, 27]. One possible hypothesis is that the protective functions of HDL particles are affected in CAD patients [28]. This would mean that measurement of HDL-C would not accurately reflect the functionality of HDL particles. Indeed, as observed in our study population, CAD patients display features of metabolic syndrome with moderate hypertriglyceridemia. In the long term, this condition alters the lipid and proteome composition of HDL particles, leading to impaired vascular protective properties [28].

IF1 was significantly and inversely associated with mortality in CAD patients independently of several established risk factors and even after further adjustments for biomarkers of cardiac diseases (NT-proBNP, hs-TnT) or clinical parameters associated to cardiovascular complications (ABI, heart rate). This indicates that IF1 level would reflect a biological process not captured by classical cardiovascular risk factors or bio-clinical variables and current biomarkers of cardiac functions. Mitochondrial IF1 has been reported to be over-expressed in cancer cells and tumors, being possibly involved in cell survival and proliferation through activation of aerobic glycolysis and reactive oxygen species signaling [29, 30]. Analysis of tumor expression of cellular IF1 in cohorts of breast cancer patients revealed its relevance as a predictive marker for clinical outcome [30]. In the present study, serum IF1 level was not associated with cancer mortality, suggesting that circulating IF1 does not reflect its mitochondrial function in metabolic reprogramming and cell survival. However, evaluation of serum IF1 in cancer epidemiology cohorts might help to better evaluate its relevance in cancer prediction and prognosis.

At present, it is only possible to hypothesize on the mechanisms linking IF1 and cardiovascular mortality and on the reciprocal relationships between circulating IF1 and HDL markers, including HDL-C, HDL particle size, and HDL-P. Bearing in mind that exogenous recombinant IF1 reduces HDL endocytosis in hepatocytes and perfused rat livers [12], circulating IF1 might actively slow down hepatic HDL holoparticle uptake in vivo, which would translate into increased levels of HDL-C, HDL-P, and HDL particle size. However, this raises the question of how reducing HDL catabolism might be beneficial against atherosclerosis. Slowing down HDL catabolism might prolong the residence time of HDL particles, enabling them to exert their beneficial anti-atherosclerotic properties (e.g., anti-inflammatory, anti-thrombotic, anti-oxidant, vasorelaxant, and cytoprotective effects) on the vascular wall. Conversely, this might not have a major negative impact on the whole process of RCT because, in humans, the final steps (liver uptake) occur not only through HDL uptake but also through low-density lipoprotein uptake following lipid transfers between lipoproteins [31]. An alternative hypothesis for the negative association between serum IF1 and CAD risk is that IF1 is released from the plasma membrane where it constitutively binds to ecto-F_1_-ATPase. Under this hypothesis, elevated IF1 might actually reflect increased functional activity of ecto-F_1_-ATPase as stimulated by apoA-I. This view is supported by recent studies reporting that endogenous IF1 is expressed at the plasma membrane of different cell lines, including hepatocytes, where it constitutively inhibits ecto-F_1_-ATPase activity [32–34]. One hypothesis is that apoA-I, which competes with IF1 for binding sites on ecto-F_1_-ATPase [12–15], contributes to the release of ecto-IF1 from ecto-F_1_-ATPase. This would subsequently promote extracellular ADP production and P2Y_13_-mediated HDL endocytosis, of which activation has been reported to be athero-protective in mice [15, 35]. In addition, on endothelial cells, stimulation of ecto-F_1_-ATPase activity by apoA-I contributes to HDL-mediated endothelial protection [13, 14]. Therefore, circulating IF1 may participate in the complex negative relationship between HDL and atherosclerosis. Further studies assessing associations between IF1 and other HDL markers, such as HDL particle size, concentration and functions, will bring new insight into the relationship between IF1 and HDL turnover, remodeling, and beneficial anti-atherosclerotic properties.

In addition to HDL markers, IF1 correlated negatively with heart rate and NT-proBNP, and positively with LVEF, suggesting that serum IF1 might also reflect myocardial function. In this context, part of circulating IF1 might originate from cardiomyocyte mitochondria. Interestingly, preconditioning experiments have demonstrated that, in ischemic conditions, IF1 is actively recruited to mitochondrial F_1_F_O_ ATP-synthase to block the massive breakdown of cellular ATP pools [15]. Accordingly, hypoxia-inducible factor 1 α was shown to regulate transcriptional levels of IF1 [36]. One could hypothesize that increased serum IF1 would indicate optimal cardiomyocyte function. Interestingly, IF1 was not correlated to hs-TnT, suggesting again that low IF1 levels would be rather related to myocardial dysfunction than to the extent of necrosis.

Besides the biological markers discussed above and the clinical variables related to CAD severity, our study identifies other individual parameters associated to mortality in CAD patients. These include lower educational level, higher tobacco consumption, and reduced physical activity, which itself is probably related to higher waist circumference. These parameters may contribute to the identification of people at high risk of cardiovascular complications. Finally, treated dyslipidemia appears to be more frequent among survivors than in patients who died. Statistical analysis revealed that the HR associated with statin administration was 0.64 (95 % CI, 0.46–0.89, *P* < 0.01, not shown). This observation is concordant with the reported beneficial effects, as regards mortality, of statins when administered early after the first acute coronary event [37, 38].

Several limitations of our study merit comment. First, the small size of the study and the number of CAD patients with incomplete data is a limitation. Indeed, 30 % of the CAD patients from the GENES cohort did not have complete biological data and these patients were thus not included in analyses. However, similar clinical and biological characteristics were measured between included and non-included patients (Additional file 1: Table S3). Second, the *GENES* study had a cross-sectional design but the yearly assessment of vital status provided a prospective frame enabling exploration of the predictive value of markers in relation with mortality. Nevertheless, the fact that other major coronary endpoints – recurrent infarction, revascularization procedure – or cardiovascular events could not be considered is a limitation of this study. Further, focusing on mortality only resulted in small numbers of “secondary events”. Finally, this study was designed only with men, which has the advantage of recording a larger number of events than in a mixed all-gender cohort, but limited the translatability of our results to women. However, although testosterone and sex hormone binding globulin levels have been reported to be positively correlated with HDL-C [39], we previously found no gender-based differences regarding the relationship between serum IF1 and HDL-C [16], suggesting that sex hormones are unlikely to impact this relationship and that the negative association of IF1 levels with mortality could also exist in women.

## Conclusions

In conclusion, our study identifies circulating IF1 as a novel HDL-related biomarker strongly and independently associated to mortality in CAD patients. This original observation will prompt new survey studies in larger all-gender prospective cohorts in order to evaluate the value added by IF1 over existing predictors and to assess its usefulness in routine clinical practice. As compared to other emerging HDL-related biomarkers that have been also proven to be better than HDL-C level, such as HDL-P and subclasses measured by NMR [40, 41] or cholesterol efflux capacity measured by cellular assays [42], measurement of IF1 by immunoassay is technically more feasible in terms of analytical requirements, accessibility, turnaround time, and traceability. In the future, IF1 measurement could be clinically useful for better prognosis in populations at high risk or in the setting of pharmacotherapy.

## Additional file

Additional file 1: Table S1. Vital status according to administered medication in coronary artery disease patients (n = 577). **Table S2.** Correlation between IF1 and HDL, measured by standard method and NMR spectroscopy in coronary artery disease patients (n = 212). **Table S3.** Comparison between coronary artery disease patients included in the study and non-included patients. (DOCX 31 kb)
