# Supplementary material for: Serum levels of mitochondrial inhibitory factor 1 are independently associated with long-term prognosis in coronary artery disease: the GENES Study
Source: BMC Med. 2016 Aug 23;14(1):125. doi: 10.1186/s12916-016-0672-9 (PMC4994300; doi:10.1186/s12916-016-0672-9)
Supplement: Additional file 1: Table S1. — Vital status according to administered medication in coronary artery disease patients (n = 577). Table S2. Correlation between IF1 and HDL, measured by standard method and NMR spectroscopy in coronary artery disease patients (n = 212). Table S3. Comparison between coronary artery disease patients included in the study and non-included patients. (DOCX 31 kb) [file 12916_2016_672_MOESM1_ESM.docx]

|  |  | Alive (n=407) | Dead (n = 170) | p^#^ | |
| --- | --- | --- | --- | --- | --- |
| Statins |  | 62.2 | 51.2 | 0.02 | |
| Fibrates |  | 8.1 | 7.7 | 0.86 | |
| Antiplatelet agents |  | 96.8 | 88.8 | 0.001 | |
| Beta-blocker agents |  | 49.4 | 32.4 | 0.001 | |
| ACE inhibitor |  | 19.9 | 32.4 | 0.002 | |
| Calcium channel inhibitor |  | 13.3 | 14.1 | 0.79 | |
| **Table S1. Vital status according to administered medication in coronary artery patients (n = 577).**  *Antiplatelet agents: acetylsalicylic acid or clopidogrel*  *ACE inhibitor: Angiotensin converting enzyme inhibitor*  ^#^*Chi2 test* | | | | |  |

|  |  | **HDL-C (g/L)** | **HDL−P (μmol/L)** | **HDL−S (nm)** |
| --- | --- | --- | --- | --- |
| **IF1 (mg/L)** |  | 0.35 (0.23-0.46)*** | 0.30 (0.17-0.42)*** | 0.21 (0.08-0.34)** |
| **HDL-C (g/L)** |  | 1 | 0.72 (0.65-0.78)*** | 0.48 (0.36-0.57)*** |
| **HDL−P (μmol/L)** |  |  | 1 | -0.04 (-0.18-0.09) |
| HDL particle size **(nm)** |  |  |  | 1 |
| **Table S2. Correlation between IF1 and HDL, measured by standard method and NMR spectroscopy in coronary artery patients (n = 212).**  *Spearman rank correlation (95% confidence interval) **p<0.01, *** p<0.001*  *IF1: inhibitory factor 1 (mean, 0.43* ± *0.13 mg/L)*  *HDL-C: HDL-cholesterol (mean, 0.41 ± 0.10 g/L)*  *HDL-P: HDL particle concentration (mean, 26.43 ± 5.5 μmol/L)*  *HDL particle size: average HDL particle size (mean, 8.87 ± 0.34 nm)* | | | | |

|  |  | Included (n=577) | Non-included  (n=257) | p |
| --- | --- | --- | --- | --- |
| Death (%) |  | 29.5 | 26.1 | 0.32 |
| Age (years) |  | 60.5 (8.0) | 59.2 (8.2) | 0.05 |
| School (years of education) |  | 9.6 (3.0) | 9.8 (3.1) | 0.27^✝^ |
| Smoking (pack year) |  | 36.6 (36.3) | 39.7 (35.4) | 0.25^✝^ |
| Alcohol (g/day) |  | 28.5 (31.0) | 28.2 (34.2) | 0.91 |
| Physical activity (high level) (%) |  | 11.6 | 12.8 | 0.68 |
| Treatment for diabetes (%) |  | 24.4 | 24.5 | 0.98 |
| Treatment for dyslipidemia (%) |  | 64.3 | 62.3 | 0.58 |
| Treatment for hypertension (%) |  | 44.9 | 41.6 | 0.39 |
| BMI (kg/m^2^) |  | 27.4 (4.0) | 27.7 (4.0) | 0.84 |
| Waist circumference (cm) |  | 98.9 (10.9) | 98.8 (10.9) | 0.82 |
| Systolic blood pressure (mm Hg) |  | 139.4 (20.9) | 140.1 (19.9) | 0.64 |
| Resting heart rate (beat/mn) |  | 63.7 (11.9) | 65.2 (12.6) | 0.10^#^ |
| Fasting glucose (mmoL/L) |  | 5.90 (2.01) | 6.16 (2.37) | 0.13^✝^ |
| Triglycerides (g/L) |  | 1.64 (0.86) | 1.87 (1.26) | 0.02^#^ |
| Total cholesterol (g/L) |  | 2.00 (0.42) | 2.04 (0.47) | 0.12 |
| LDL-C (g/L) |  | 1.24 (0.37) | 1.26 (0.43) | 0.39 |
| HDL-C (g/L) |  | 0.43 (0.12) | 0.43 (0.12) | 0.91 |
| Lp(a) ≥0.30 vs <0.30 g/L (%) |  | 55.4 | 50.2 | 0.40 |
| eGFR <30 mL/min (%) |  | 1.7 | 1.7 | 1.00^‡^ |
| hs-CRP ≥ 5 mg/L vs < (%) |  | 55.3 | 46.6 | 0.03 |
| hs-TropT (pg/mL) |  | 161 (413) | 105 (317) | 0.001^✝^ |
| NT-proBNP (pg/mL) |  | 642 (1454) | 643 (1558) | 0.99^✝^ |
| ABI ≤0.9 (%) |  | 34.8 | 29.4 | 0.13 |
| LVEF ≥ 50% (%) |  | 73.5 | 72.4 | 0.74 |
| Gensini score |  | 47.8 (40.6) | 44.3 (39.6) | 0.31^#^ |
| Duration of coronary artery disease (months) |  | 40.5 (62.3) | 47.4 (74.3) | 0.19^✝^ |
| **Table S3. Comparison between CAD patients included and patients non included.**  *Data are expressed in mean (SD) or %.*  ^#^*log transformed data,* ^✝^*Wilcoxon-Mann-Withney test,* ^‡^*Fischer's exact test.*  *BMI: Body Mass Index,*  *eGFR: estimated Glomerular Filtration Rate,*  *hs-CRP: high-sensitivity C-Reactive Protein,*  *hs-TropT: high-sensitivity cardiac troponin T,*  *NT-proBNP: N-terminal pro-brain natriuretic peptide,*  *ABI: Ankle-brachial index,*  *LVEF: Left Ventricular Ejection Fraction***.** | | | | |
